# Nocturnal Lagophthalmos and Sleep Quality in Patients with Dry Eye Disease

**DOI:** 10.3390/life10070105

**Published:** 2020-07-04

**Authors:** Aya Takahashi, Kazuno Negishi, Masahiko Ayaki, Miki Uchino, Kazuo Tsubota

**Affiliations:** 1 Department of Ophthalmology, Keio University School of Medicine, Tokyo160-5852, Japan; uchinomiki@yahoo.co.jp (M.U.); tsubota@z3.keio.jp (K.T.); 2 Department of Ophthalmology, Tokyo Dental College Ichikawa General Hospital, Chiba 272-8513, Japan; 3 Otake Clinic Moon View Eye Center, Kanagawa 242-0001, Japan; 4 Tsubota Laboratory, Inc., Tokyo 160-8582, Japan

**Keywords:** nocturnal lagophthalmos, dry eye disease, sleep quality

## Abstract

Nocturnal lagophthalmos (NL) refers to the inability to close the eyelids during sleep, which is known to affect dry eye disease (DED) symptoms and sleep quality. This study aimed to evaluate the prevalence of NL and sleep quality in DED patients. We launched a survey website to recruit 2000 Japanese. The participants were asked to answer a questionnaire about DED, sleep quality, and happiness. Participants were divided into two groups according to the presence of DED, and responses were compared between the groups. The DED group was comprised of 890 subjects (44 ± 13.8 years, 359 males) and women were predominant (*p* < 0.001). Sleep duration was significantly shorter (*p* = 0.008), sleep latency was longer (*p* < 0.001), and sleep efficacy was worse compared with the non-DED group (*p* < 0.001). Furthermore, people belonging to the DED group were more frequently working night shifts (*p* < 0.001). NL was more prevalent in the DED group (*p* = 0.007). Logistic regression analysis showed that NL correlated with younger age, symptomatic DED, and eye symptoms upon waking. The current study suggested that NL was associated with worsened DED symptoms and poor sleep quality. Preventative eye care for lagophthalmos before and during sleep may be helpful for DED and sleep quality.

## 1. Introduction

**Figure 1:**
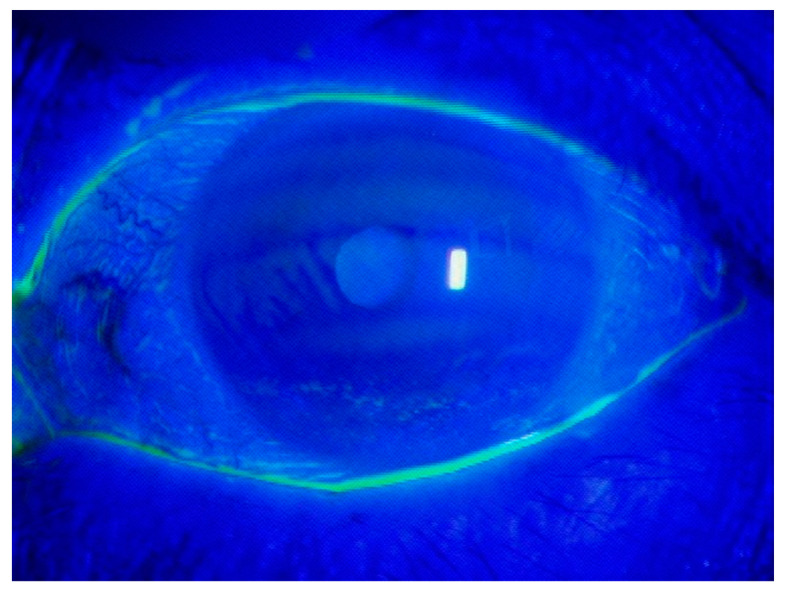
Clinical microscopic photograph with vital corneal staining. This patient is a 65-year-old woman who presented at the clinic complaining of eye irritation in the morning.

Nocturnal lagophthalmos (NL) refers to the inability to close the eyelids during sleep. The involuntary closure of eyelids during sleep provides protection to the ocular globe from mechanical insults [1]. Lack of this phenomenon can cause tear film dysfunction and worsen dry eye disease (DED) symptoms. For example, the exposed corneal area often shows an inferior horizontal punctate epithelial keratitis in the morning (Figure 1). DED is a multifactorial disease of the tear film and is known to be increasing in prevalence worldwide. Its symptoms affect the activities of daily life and patients’ quality of life [2,3,4]. The prevalence of the disease is 5–50% and tends to be higher in women and members of the Hispanic and Asian races, compared to Caucasians [5].

Although clinical ophthalmologists often encounter patients with severe inferior epithelial keratitis early in the morning, detailed symptomatology, NL, and sleep quality have not been comprehensively evaluated. NL could be associated with symptomatic ocular surface disorders, and those conditions should be evaluated. Understanding the prevalence of NL and its implications for DED and sleep quality may provide a clinical rationale for the nighttime treatment of symptomatic patients.

This study aimed to investigate the prevalence of NL and sleep quality in DED patients, and to determine their relationship in order to propose better eye care for DED patients with NL.

## 2. Methods

### 2.1. Patient Recruitment

We hired MediProduce Incorporated (Tokyo, Japan), a company that is certified in the protection of personal information, and launched a survey website to recruit 2000 people, adjusted for age and gender. All subjects older than 20 years who used the web survey panel (Ef Press Incorporated, Tokyo, Japan) were asked to participate in this study. Among 1,700,000 panels, participants who used a visual display terminal were randomly selected and invitation mails were sent without introducing the aim of the study. The first 2000 participants who satisfied requirements were enrolled in this study. The study took place from 4 April to 13 April 2016. Participants received no money, but instead received reward points, which could be used only on the panel website, as compensation. The age groups were: twenties, thirties, forties, fifties, and sixties.

### 2.2. Ethics Statements

The research was conducted in accordance with the ethical principles of the Declaration of Helsinki and it was approved by the Institutional Review Board of Keio University (permit no. 20150399, 6 May 2015). Written informed consent, including approval for the use of information collected during the study, was obtained from the participants through the survey website.

### 2.3. Questionnaire

The participants were asked to answer 17 questions on physical information, symptoms and history of DED diagnosis, lifestyle, sleep quality, and subjective happiness (Table 1). We evaluated age, gender, body mass index (BMI, kg/m^2^) (Q1 and 2), sleep duration (S8, 9 and 10), sleep latency (S10), sleep efficacy (the ratio of sleep duration to time in bed) (S8, 9 and 10), subjective sleep quality (S7), subjective happiness (L17), time spent outside per day (L16), frequency of working night shifts (L15), presence of NL (S11), presence of dryness of mouth (D6), and a number of DED-related symptoms (M13, answers 3 to 10). Symptoms were also analyzed in detail (M13): none (answer 1), difficulty opening eyes (3), foreign body sensation (4), eye pain (5), discharge (6), tears (7), dryness (8), and photophobia (9).

### 2.4. Data and Statistical Analyses

The subjects were divided into two groups according to the presence of NL. Subjects who answered 1 for question S11 were categorized as the NL (+) group, and those who answered 2 were categorized as the NL (−) group. We did not include subjects who answered 3 (not sure) in the analysis. We also compared groups with and without DED based on the answers to the validated short questionnaire (dryness and irritation of the eyes, and former diagnosis of DED [6]). We defined DED as having a previous diagnosis of DED (D5) and/or having both dryness (answer 1 or 2 on D3) and foreign body sensation (answer 1 or 2 on D4) in the eyes. Each variable was compared between the groups. Logistic regression analysis was performed using the presence of NL as a dependent valuable. 

Where appropriate, data are given as mean ± standard deviation (SD). Given that the data were not normally distributed, we applied Student’s *t*-test, the Wilcoxon signed rank test, the chi-squared test, the Mann–Whitney U test, and age-and-gender-adjusted multivariate logistic regression analysis for statistical analysis in Stata, software version 16. A *p*-value of <0.05 was considered statistically significant.

## 3. Results

Of the 2000 subjects, 90 (4.5%) had NL (40.5 ± 13.8 years, 42 males) and 1174 (58.7%) were without NL (44.8 ± 13.9 years, 574 males), while 736 subjects answered that they were not sure about their NL phenomenon (44.9 ± 13.7 years, 382 males) and 890 (44.5%) were assigned to the DED group (44 ± 13.8 years, 359 males).

In the NL group, subjects were significantly younger, had more frequent night shifts, and had more symptoms related to their eyes upon waking. Subjects with difficulty opening their eyes, foreign body sensation, and eye pain in the morning were likely to have NL (Table 2).

**Figure 2:**
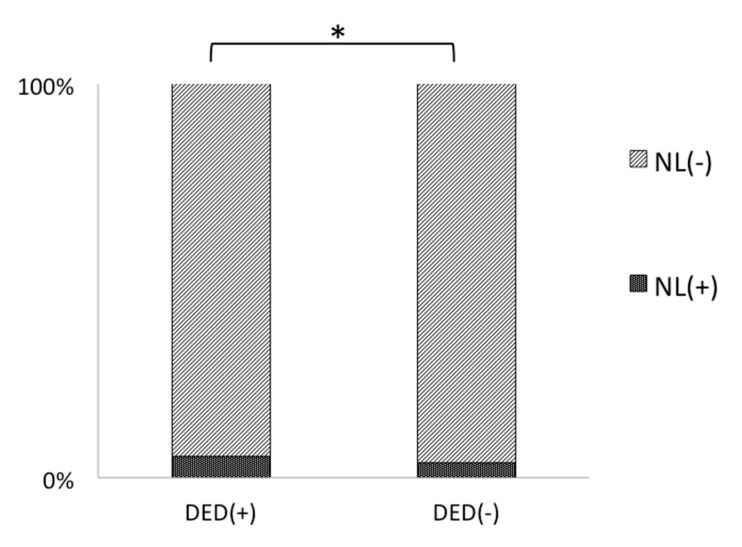
Nocturnal lagophthalmos was more prevalent in the group with dry eye disease than in the group without. * *p* < 0.05 *t*-test.

In the DED group, there were more women than men (*p* < 0.001). Sleep duration was significantly shorter (*p* = 0.008), sleep latency was longer (*p* < 0.001), and sleep efficacy was worse compared with the non-DED group (*p* < 0.001). Furthermore, awakening during sleep was more prevalent in the DED group (*p* < 0.001). NL was also more prevalent in the DED group (*p* = 0.007; Table 3, Figure 2).

Logistic regression analysis produced robust results in the comparison between the two groups stratified by NL (Table 3), and it revealed that NL was significantly correlated with younger age, symptomatic DED, and more eye symptoms in the morning. Subjects with difficulty opening their eyes and eye pain were especially likely to have NL (Table 4).

## 4. Discussion

**Figure 3:**
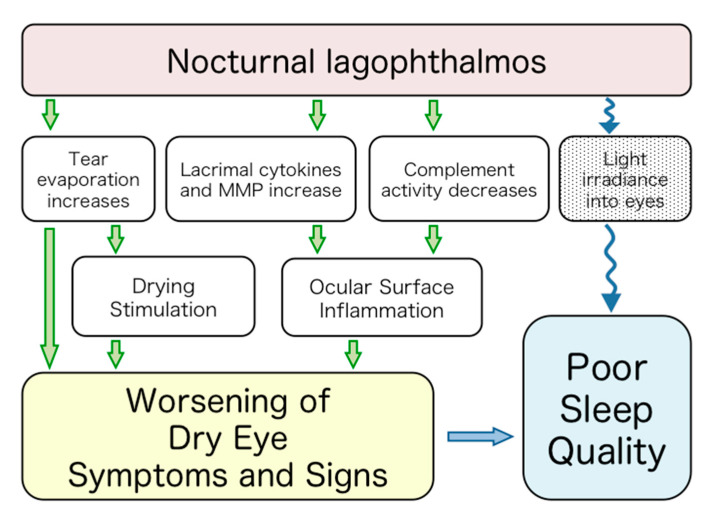
Hypothesis of the effects of nocturnal lagophthalmos on dry eye disease and sleep quality.

The current study suggested that NL was associated with DED and poor sleep. Herein, we propose the hypothesis that NL could be a hidden risk factor for DED and poor sleep (Figure 3). NL can be caused by facial nerve paralysis or facial trauma, but it is also a common phenomenon observed in otherwise-healthy individuals [1]. Reported underlying causes include alcohol consumption and the use of hypnotic medication [7]. NL was also documented as a hereditary congenital abnormality in *System of Ophthalmology* [8], and Sturrock et al. suggested the possibility of a genetic factor in some patients [9]. In this study, 4.5% of the subjects had a history of physiological NL, which was concordant with the previously reported prevalence in the Chinese population [10], whereas NL was found in 1.4% of the Caucasian population [11]. Mueller suggested that the phenomenon was common in Amharic people in Ethiopia due to their feature of large eyes [12].

McNab suggested that NL patients tend to be poor sleepers since the closing of the eyes during sleep may help induce and maintain sleep by reducing visual input into the cerebral cortex, as well as helping protect the ocular surface during sleep [13]. Long wavelength light transmits through closed eyelids and can lead to circadian sleep disorders (9% transmittance at 630 nm wavelength) [14]. This relationship is likely to be enhanced with partly opened eyes. Indeed, we observed a tendency of having poor subjective sleep quality in subjects with NL (*p* = 0.07). NL may adversely affect sleep quality by 1) increasing the intensity of light stimuli and 2) changing the ocular surface environment, mainly affecting the tear film, which induces DED symptoms. The present results further support larger studies with a nationwide cohort to investigate the presumed effects of NL shown in the schematic presentation in Figure 3. 

Tear functional change at night in patients with NL may result in a deterioration of DED symptoms. Closed-eye tears contain a functional complement system and regulatory compounds, and are activated while eyes are closed during sleep [15,16,17]. When eyes are open during sleep, the complement activity decreases, so we can hypothesize that the function of the regulatory system would diminish. Recent studies on DED revealed an inflammatory process and associated pathogenesis [18]. 

There are various ways to treat NL and its symptoms. The most effective and simple is using ophthalmic ointment at night and artificial tears or eye drops treating DED symptoms. Taping the eyelid closed during sleep has also proved helpful [19]. The present results showed that younger age and shift work were notable risk factors for NL. We assume that increased sympathetic activity might be related to insufficient closure of the eyelids during sleep by increasing stimulation of the Muller muscle [20]. Decreasing sympathetic activity and increasing parasympathetic activity at night, for example with hot eye masks [21], aroma therapy [22], and taking a bath [23], would help prevent NL. These countermeasures for NL could substantially improve sleep quality. The future outlook of NL treatment would include careful examination of the underlying disease, proper instruction on alcohol consumption to patients, and re-examining patients’ oral medications, including hypnotic agents. Since DED and sleep disorders are possibly related to NL mutually, treatment of DED or of sleep disorders might be helpful for treating NL. For genetically associated NL, neurosurgery and oculofacial surgery may be indicated in addition to medical measures according to severity and disability.

The diagnosis of NL can be challenging because there are no changes on the eyelids during the day. When patients with certain symptoms undergo an ophthalmic examination, findings such as punctate keratoplasty and insufficient closure of eyes when they blink involuntarily can easily be ignored. This is one of the reasons that the epidemiology and effects of NL on health are not well known.

The present results were compatible with previous reports revealing that women are more likely to have DED compared to men [24] and that people with DED had poor sleep quality, especially DED-symptom-related sleep quality [25,26,27,28,29]. The results might provide additional evidence for a clinical association between DED and sleep, and help serve DED patients better.

We acknowledge several limitations of this study. First, the relatively low frequency of NL may be a limitation on analyzing the data. Nevertheless, this study successfully captured the close relationship between NL and DED to propose new insights into the diagnosis and management of DED. Second, clinical data would confirm the current results, since DED diagnosis relied on self-reported questionnaires, although we used a questionnaire that had been suitably validated to evaluate the prevalence of DED. Another limitation of using a self-administered questionnaire, which might have introduced biases, pertained to possible misunderstanding regarding the questions on medication use and the subjects’ health status. This study only includes a self-screening evaluation, which might be a limitation. However, a larger sample size should overcome this limitation, and this study design may be sufficient to demonstrate the association of DED, sleep quality, and NL. The current study showed a correlation between NL and sleep quality. For further elucidating a causal relationship, we suggest a systematic evaluation, including ophthalmic examination and physical evaluation.

## 5. Conclusions

The current study suggests that NL is associated with DED and sleep quality. Managing lagophthalmos and related symptoms may help improve DED and sleep quality. Further studies would contribute to establishing etiology-based classification, pathology, preventive measures, and effective treatments for NL.

## Figures and Tables

**Table 1:** The questionnaire used in the study.

Question	Possible Answers
1. Height	m
2. Weight	kg
**Dry Eye**	
D3. “How often do you feel dryness in your eye?”	1: constantly, 2: sometimes, 3: rarely, 4: never
D4. “How often do you have a sensation of a foreign body in your eye?”	1: constantly, 2: sometimes, 3: rarely, 4: never
D5. “Have you been diagnosed as having DED?”	1: yes, 2: no
D6. “Do you have a dry mouth?”	1: constantly, 2: sometimes; 3: rarely, 4: never
**Sleep**	
S7. “How would you rate the quality of your sleep?”	1: great, 2: good, 3: average, 4: not good, 5: poorly
S8. “At what time do you get up during weekdays?”	Time
S9. “At what time do you go to bed during weekdays?”	Time
S10. “How long does it take you to fall asleep after you go to bed?”	Duration (minutes)
S11. “Are your eyes open during sleep?”	1: yes, 2: no, 3: not sure
**Morning eye symptom**	
M12. “Do you wake up to eye pain during sleep?”	1: never, 2: once a week, 3: twice to four times a week, 4: every night
M13. “How do your eyes feel when you wake up?”	1: good, 2: not good, 3: have difficulty opening, 4: feel foreign body sensation, 5: feel pain, 6: eye discharge, 7: lacrimation, 8: dry, 9: sensitive to light, 10: others, multiple answers allowed
M14. “Do you take eye drops after waking or for sleeping?”	1: at waking, 2: at sleeping, 3: neither
**Lifestyle**	
L15. “How often do you have a night shift?”	Time per month
L16. “How long do you spend outside during daytime?”	1: less than one h, 2: 1 h, 3: 2 h, 4: 3 h, 5: 4 h, 6: 5 h, 7: 6 h, 8: 7 h, 9: 8 h, 10: 9 h, 11: more than 10 h
L17. “How would you rate your happiness?”	7–1: happiest–unhappiest

**Table 2:** Characteristics of the study population according to nocturnal lagophthalmos (NL) category.

	NL (+) *n* = 90 (5%)	NL (−) *n* = 1174 (59%)	*p*-Value
Age (years)	40.5 ± 13.8	44.8 ± 13.9	0.004 *
Gender (male/female)	42/48	574/600	0.68
BMI	22.5 ± 3.8	22.2 ± 3.7	0.50
**Sleep Parameters**			
Sleep latency (minutes)	26.4 ± 27.5	22.3 ± 22.1	0.10
Sleep efficacy (%)	93.3 ± 6.5	94.3 ± 5.7	0.13
Sleep duration (minutes)	370 ± 64	377 ± 73	0.37
Subjective sleep quality	2.83 ± 1.07	2.63 ± 1.02	0.07
Subjective happiness	4.2 ± 1.4	4.4 ± 1.5	0.29
Outdoor time (hours)	3.5 ± 2.3	3.1 ± 2.3	0.16
Night shift (times/month)	1.76 ± 5.4	0.70 ± 0.5	0.002 *
Number of symptoms of eyes upon waking	1.09 ± 1.3	0.71 ± 0.92	<0.001 *
**Symptoms in Detail**			
None	39 (43.3%)	592 (50.5%)	0.189
Difficulty opening eyes	13 (14.4%)	63	<0.001 **
Foreign body sensation	18	95	<0.001 **
Eye pain	6	29	0.020 **
Discharge	22	276	0.85
Tears	8	57	0.10
Dryness	8	103	0.97
Photophobia	3	98	0.09

NL: nocturnal lagophthalmos, BMI: body mass index. * *p* < 0.05 *t*-test, ** *p* < 0.05 chi-squared test, *** *p* < 0.05 Mann–Whitney U test.

**Table 3:** Characteristics of the study population according to dry eye disease (DED) category.

	DED (+) *n* = 890 (45%)	DED (−) *n* = 1110 (56%)	*p*-Value
Age (years)	44.0 ± 13.8	45.2 ± 13.9	0.05
Gender (male/female)	359/531	641/469	<0.001 **
BMI (kg/m^2^)	22.2 ± 4.0	22.4 ± 3.7	0.26
**Sleep Parameters**			
Sleep latency (minutes)	25.5 ± 24.6	21.6 ± 21.3	<0.001 *
Sleep efficacy (%)	93.4 ± 6.5	94.5 ± 5.7	<0.001 *
Sleep duration (minutes)	368 ± 74	377 ± 76	0.008 *
Subjective sleep quality	2.91 ± 1.02	2.60 ± 1.04	<0.001 ***
Subjective happiness	4.2 ± 1.5	4.3 ± 1.5	0.04 *
Outdoor activity time (hours)	3.3 ± 2.5	3.1 ± 2.4	0.05
Night shift (times/month)	0.96 ± 3.2	0.63 ± 2.9	0.02 *
Nocturnal lagophthalmos	49 (5.4%)	41(3.7%)	0.007 *
Dryness of mouth	398 (44.7%)	415 (37.4%)	0.001 **
Number of symptoms of eyes upon waking	1.24 ± 1.1	0.47 ± 0.43	<0.001 *
**Symptoms in Detail**			
None	225 (24.7%)	687 (75.3%)	<0.001 **
Difficulty opening eyes	90	36	<0.001 **
Foreign body sensation	171	32	<0.001 **
Eye pain	14	49	<0.001 **
Discharge	301	204	<0.001 **
Tears	69	45	<0.001 **
Dryness	145	41	<0.001 **
Photophobia	102	56	<0.001 **

DED: dry eye disease, BMI: body mass index. * *p* < 0.05 *t*-test, ** *p* < 0.05 chi-squared test, *** *p* < 0.05 Mann–Whitney U test

**Table 4:** Risk factors for having NL.

Variables	Odds Ratio	95% Confidence Interval	*p*-Value
Age #	0.97	0.96–0.99	0.005 *
Gender (1: male, 0: female) #	0.94	0.58–1.35	0.78
BMI (kg/m^2^)	1.03	0.97–1.09	0.30
DED (1: (+), 0: (−))	1.75	1.12–2.71	0.01 *
Sleep parameters			
Sleep latency (minutes)	1.00	0.99–1.01	0.55
Sleep efficacy (%)	1.00	0.96–1.04	0.94
Sleep duration (minutes)	1.00	0.99–1.00	0.11
Subjective sleep quality	1.16	0.94–1.43	0.16
Subjective happiness	1.07	0.92–1.26	0.35
Outdoor time	1.06	0.98–1.15	0.16
Night shift (times/month)	0.98	0.91–1.06	0.62
Dryness of mouth (1: (+), 0: (−))	1.00	0.65–1.56	0.99
Number of symptoms of eyes upon awakening	1.23	1.03–1.47	0.04 *
**Symptoms in detail**			
Difficulty opening eyes (1: (+), 0: (−))	1.99	1.00–3.95	0.049 *
Foreign body sensation (1: (+), 0: (−))	1.00	0.47–2.14	1.00
Eye pain (1: (+), 0: (−))	2.71	1.09–6.75	0.03 *
Discharge (1: (+), 0: (−))	1.37	0.85–2.19	0.20
Tears (1: (+), 0: (−))	0.82	0.29–2.30	0.70
Dryness (1: (+), 0: (−))	1.73	0.94–3.19	0.08
Photophobia (1: (+), 0: (−))	1.11	0.52–2.37	0.80

NL: nocturnal lagophthalmos, BMI: body mass index, DED: dry eye disease. # Multivariate logistic regression analysis was performed adjusted for age and gender. * *p* < 0.05.
